# Study on the flammability, thermal stability and diffusivity of polyethylene nanocomposites containing few layered tungsten disulfide (WS_2_) functionalized with metal oxides

**DOI:** 10.1039/c8ra01527a

**Published:** 2018-04-09

**Authors:** K. Wenelska, K. Maślana, E. Mijowska

**Affiliations:** Nanomaterials Physicochemistry Department, Faculty of Chemical Technology and Engineering, West Pomeranian University of Technology, Szczecin Piastow 45 70-311 Szczecin Poland kwenelska@zut.edu.pl

## Abstract

In this work, exfoliated tungsten disulfide (WS_2_) functionalized with metal oxides as a filler of polyethylene (PE) was used. An efficient exfoliation procedure resulted in the synthesis of 7–9 layered flakes of WS_2_. Flakes of exfoliated WS_2_ were functionalized by iron oxide and nickel oxide nanoparticles, respectively. The nanomaterials were mixed with polyethylene by extrusion. Methods such as Transmission Electron Microscopy (TEM), Atomic Force Microscopy (AFM), X-Ray Diffraction (XRD) or Thermogravimetric Analysis (TGA) were used to characterize the materials. Flame retardant properties were investigated by microcalorimetry. Comparing the obtained values of heat released during combustion, it can be observed that the addition of fillers reduces flammability significantly compared to neat polyethylene. It is revealed that this composite can provide a certain physical barrier and inhibit the diffusion of heat and gaseous products during combustion. Thermogravimetric analysis of composites showed increased thermal stability with addition of nanofillers and reduction of carbon monoxide generation in the whole range of the nanofiller addition (from 0.5 to 2 wt% in PE). Results suggested that the composite with Ni_2_O_3_ could endow the best flame retardance for PE. The peak heat release rate of this sample with 2 wt% nanofiller was reduced to 792 W g^−1^ (1216 W g^−1^ for PE), and the total heat release was decreased to 39 kJ g^−1^ (47 kJ g^−1^ for PE). A very significant increase in thermal conductivity for all composites was observed as well.

## Abstract 2

In this work, exfoliated tungsten disulfide (WS_2_) functionalized with metal oxides as a filler of polyethylene (PE) was used.

## Graphical abstract

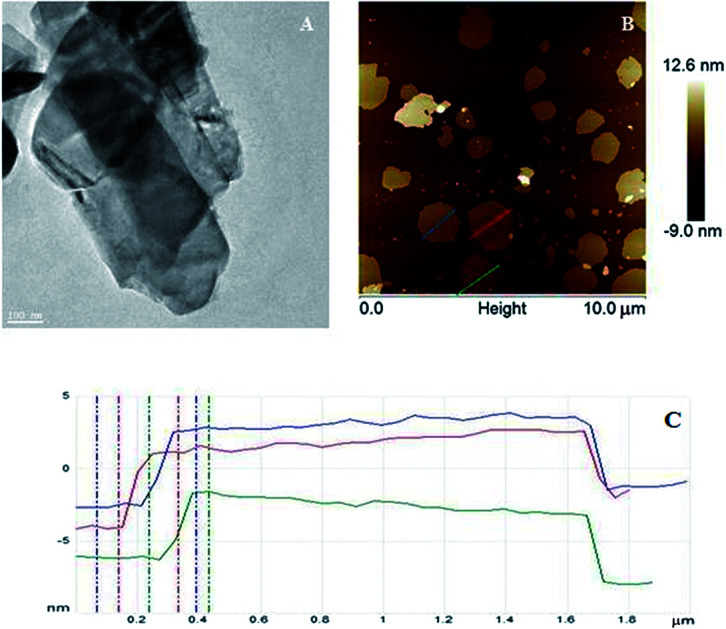

## 1. Introduction

The discovery of graphene and its superior properties have focused research interest on other two-dimensional (2D) groups of materials like hexagonal boron nitride (h-BN) and transition metal dichalcogenides (TMDs).^1^ A monolayer of TMD, the general chemical formula of which is MX_2_, consists of a metal atom (M = Mo, W) sandwiched between two chalcogenide atoms (X = S, Se)^3^ and it has a thickness between 6 and 7 Å.^2^ Depending on the coordination number and oxidation state of the metal atoms, TMDs can be metallic, semimetallic or semiconducting.^4^ These materials exhibit a layered structure^5^ with weak interlayer van der Waals forces which allow easy exfoliation.^1,3^ Unlike graphene, the band gap of these materials, which due to a reduced number of sheets, changes from an indirect band gap to a direct band gap.^1,6–9^ Therefore, these materials can have many new applications *e.g.* in electronics, optoelectronics,^3,8^ catalysis, energy storage and sensing.^3,10^

Tungsten disulfide (WS_2_) is one of the most popular compound of semiconducting TMS's. The band gap of monolayer of WS_2_ is 2.1 eV, while in bulk is 1.3 eV and that results in enhancement of photoluminescence.^11,12^ This compound exhibit trigonal prismatic structure. Mechanically exfoliated atomically thin sheets of WS_2_ were shown to exhibit high in-plane carrier mobility and electrostatic modulation of conductance similar to MoS_2_.^13^ Many techniques have been reported to obtain atomically thin layers WS_2_ like mechanical exfoliation,^13^ chemical exfoliation^4,14^ and chemical vapor deposition.^15^ WS_2_ nanosheets have broad applications in optoelectronics.^12,16–20^

Recently this group of materials has attracted increased attention in the field of nanocomposites fillers, due their graphene-like properties such as high thermal and mechanical properties.^21,22^ Polymeric materials are widely used in the most important industries. However, they are known for high fire risk and most of them combust with emission toxic gases. It is important to modify these materials to reduce their flammability. There are three typical strategies to achieve that: use of inherently flame retardant polymers,^21^ flame retardants,^22^ and surface treatment/coating.^23^ Usually small amount of nanofiller improves thermal properties of composites. WS_2_, as a typical layered inorganic material, is expected to disperse and exfoliate in polymers and it results in the physical barrier formation which inhibits the diffusion of heat and the decomposition of polymer products. So it is reasonable that WS_2_ may improve the thermal stability, mechanical properties and fire resistance of polymer composites.^24^ To the best of our knowledge there is a gap in the scientific reports on the potential of few layered WS2 functionalized with metal oxides as a nanofiller of polymers used for flame retardancy.

Polyethylene is one of the most common polymer used in everyday object in our houses. However, its flammability and toxicity during the fire are the key motivations to perform the proposed study.

In this work we present a technique of liquid exfoliation of tungsten disulfide nanosheets and its functionalization with metal oxides (nickel and iron). We use these materials as fillers in polyethylene nanocomposites and investigate their flame retardants properties by pyrolysis combustion flow calorimeter (PCFC) and thermal conductivity by xenon flash method.

## 2. Experimental

### 2.1 Reagents

Bulk tungsten(iv) disulfide (WS_2_, powder 99.9%), Nickel(ii) acetate (Ni(CH_3_COO)_2_·4H_2_O, powder <99%), Iron(ii) acetate (Fe(CH_3_COO)_2_, powder 96%) polyethylene (*d* = 0.92 g mL^−1^, powder) were obtained from Sigma-Aldrich and cetyltrimethylammonium bromide (CTAB) was purchased from AppliChem.

### 2.2 Characterization

The structures of materials before and after exfoliation/functionalization were studied by Transmission Electron Microscopy (Tecnai F20-based at 200 kV accelerating voltage). Atomic Force Microscopy (AFM NTEGRA Aura (NT-MTD) microscope) provide information about thickness and number of layers of WS_2_. X-ray diffraction (XRD) Philips X'Pert PRO X-ray diffractometer witch Cu Kα radiation was employed to identify phase identification of the samples. Thermogravimetric analysis (TGA) was carried out using a SDT Q 6000 thermoanalyzer instrument (TA Instruments Inc.) under air flow of 100 mL min^−1^. The samples with mass of about 5.0 mg were heated from room temperature to 900 °C at a linear heating rate of 10 °C min^−1^. During heating the sample in thermobalance, the generated gases were analyzed *in situ* by Quadrupole Mass Spectrometer QMS 422. All PE and its composites were fabricated using a twin screw extruder (Zamak Mercator EHP 2x12). Micro Calorimeter (FAA MICRO Calorimeter) was used to investigate the flammability properties of PE nanocomposites. Samples of about 2.0 mg were heated in air atmosphere (80% of nitrogen and 20% of oxygen) at a constant heating rate of 1 °C s^−1^ from room temperature to 700 °C. This method allowed to determinate parameters such as heat release rate (W g^−1^), heat release capacity (J g^−1^ K^−1^), total heat release (kJ g^−1^). The thermal diffusivity of the composites was measured *via* xenon flash technique using Linseis XFA 300 laser flash apparatus.

### 2.3 Exfoliation of WS_2_

Few-layered WS_2_ flakes were synthesized by liquid exfoliation of bulk WS_2_. First, 1 g of bulk WS_2_ and cetyltrimethylammonium bromide (0.5 g) were mixed, dissolved in distilled water and sonicated for 3 h. Then, the mixture was centrifuged at 10 000 rpm for 0.5 h. After that, the solution was washed few times with distilled water and dried 24 h in 60 °C.

### 2.4 Functionalization of WS_2_

Two samples of WS_2_ modified by metal oxide nanoparticles (WS_2__Ni_2_O_3_ and WS_2__Fe_2_O_3_, respectively) were prepared according to the following procedure: 0.5 g of WS_2_ and 0.5 g nickel(ii) acetate tetrahydrate (product referred to as WS_2__Ni_2_O_3_) and iron(ii) acetate (product referred to as WS_2__Fe_2_O_3_), respectively, were dispersed in 250 mL of ethanol and sonicated for 3 h. Afterwards, the mixtures were stirred for next 24 h. Finally, the samples were dried in high vacuum at 440 °C for 10 min.

### 2.5 Preparation of nanocomposites

Polyethylene (PE) was used as the polymer matrix. The content of MoS_2_, MoS_2__Ni_2_O_3_ and MoS_2__Fe_2_O_3,_ were 0.5%, 2%, 3%, respectively. The composites were prepared by extrusion molding at a temperature of 120 °C.

## 3. Results and discussion

**Fig. 1:**
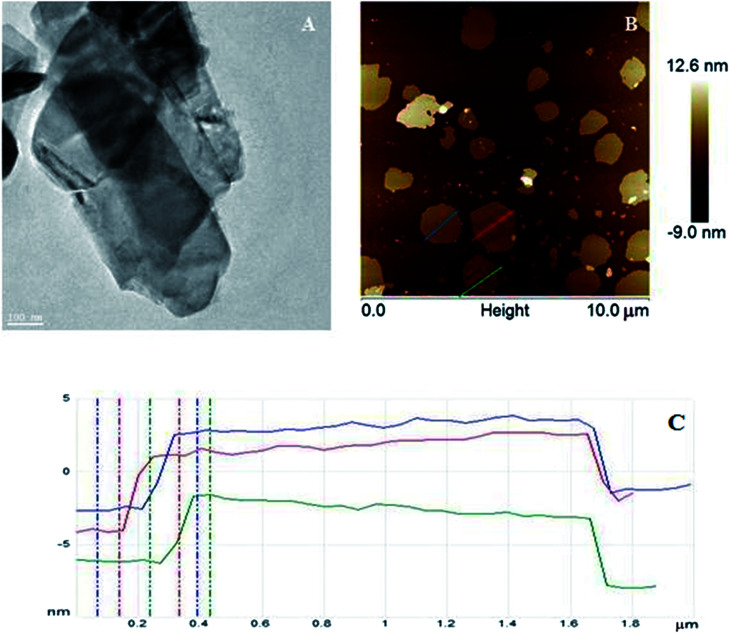
(A) TEM image, (B) AFM image and (C) high profile of exfoliated WS_2_.

The morphologies of WS_2_ after exfoliation investigated by TEM (Fig. 1A) and AFM (Fig. 1B) microscopy. Fig. 1 shows that WS_2_ was successfully exfoliated from bulk to few nanosheets. Tapping-mode of AFM was used to determinate the size and thickness of exfoliated WS_2_. The AFM samples were prepared by dropping a few drops of exfoliated WS_2_ dissolved in ethanol on silica wafer and left to evaporate the solvent. AFM analysis shows that exfoliated WS_2_ had diameter ∼0.74–1.4 μm and thickness about 4.6–5.9 nm which corresponds to 7–9 layers of WS_2_.

**Fig. 2:**
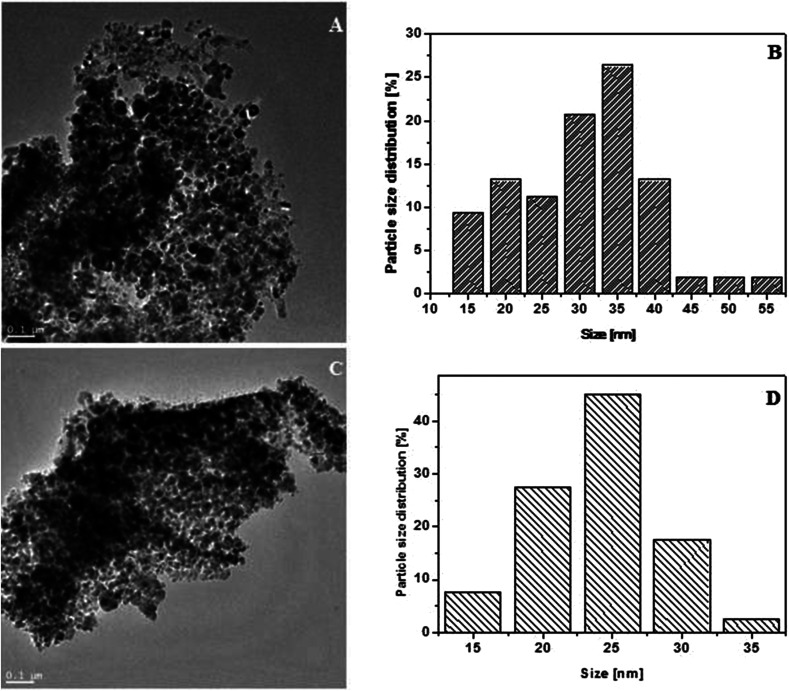
TEM images of WS_2__Fe_2_O_3_ (A) WS_2__Ni_2_O_3_ (C) and particle size distribution of WS_2__Fe_2_O_3_ (B) and WS_2__Ni_2_O_3_ (D).

After exfoliation the product was functionalized with nickel and iron compounds. The morphology and structure was characterized by TEM. Fig. 2A and C shows, that metal particles have covered the whole surface of WS_2_, what proves very good dispersion of metal oxides on WS_2_ surface. Particle size distribution of iron oxide (Fig. 2D) and nickel oxide (Fig. 2B) was estimated basing on ∼100 of particles pictured in TEM. Detailed analysis reveals very uniform diameters of samples. Particle size distribution of the iron compound and nickel compound is ∼35 and ∼25 nm, respectively.

**Fig. 3:**
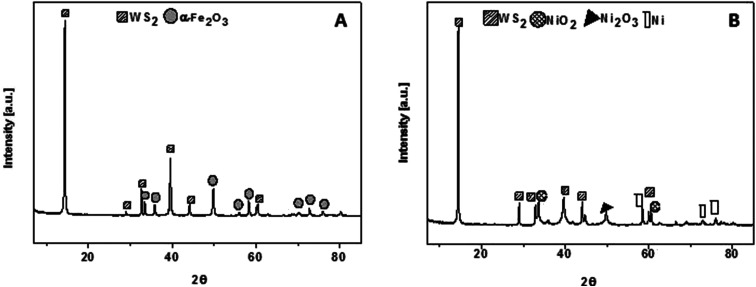
XRD pattern of (A) WS_2__Fe_2_O_3_ and (B) WS_2__Ni_2_O_3_.

The composition of WS_2__Fe_2_O_3_ and WS_2__Ni_2_O_3_ were examined by powder X-ray diffraction, as it is demonstrated in Fig. 3. In both patterns one intense pick at value 2*θ* 14° is present. This peak is assigned to WS_2_. Few peaks with lower intensity at 2*θ* value 29°, 39.5°, 44° and 60° are also associated with pristine WS_2_. Fig. 3A shows XRD pattern of WS_2__Fe_2_O_3_. Relatively strong peak is present at 50 °C. This signal belongs to iron(iii) oxide. Peaks at 33.5°, 36°, 56°, 58°, 70°, 72° and 75° are consistent with the data for the α-Fe_2_O_3_. Fig. 3B presents XRD pattern of WS_2__Ni_2_O_3_. Besides peaks which correspond to WS_2_, there are peak, as well a 2*θ* value 34° and 60° assigned to NiO_2_. Peak at 49.5° is associated with presence of Ni_2_O_3_ phase. There are also peaks of metallic nickel (peaks at 2*θ* values 58.5°, 72° and 75°). The above analysis proves that WS_2_ was successfully functionalized with nickel and iron oxides, respectively.

**Fig. 4:**
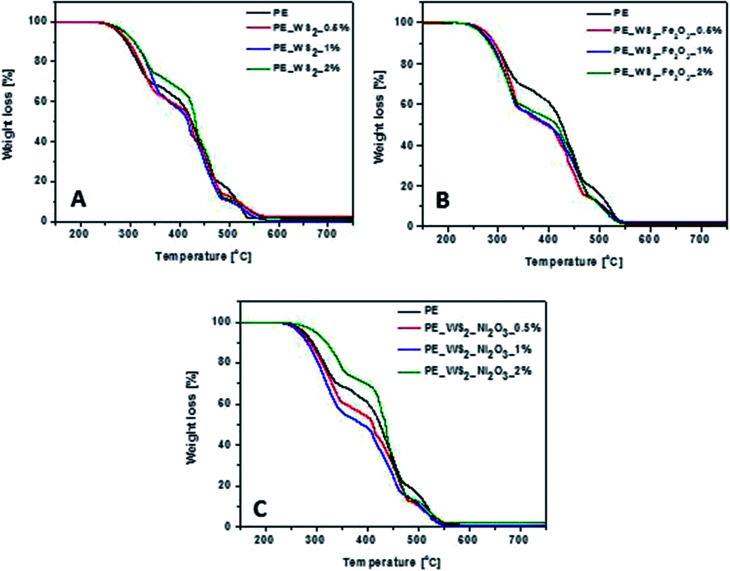
TGA profile of nanocomposites: (A) PE_WS_2_, (B) PE_WS_2__Fe and (C) WS_2__Ni.

TGA was performed to investigate the general thermal stability of PE and WS_2_ nanocomposites. Fig. 4 shows thermograms of PE and PE_WS_2_ nanocomposites. The corresponding TGA data are presented in Table 1. The temperature where the weight loss is 10 wt% is denoted as *T*_0.1_ and the temperature there half of the sample was loss is *T*_0.5_. Char yield is the weight percent obtained at the end of pyrolysis. Thermal stability of composites has decreased for most samples. The most significant drop was observed for composite PE_WS_2__Ni_2_O_3__2% for which the temperatures of *T*_0.1_ and *T*_0.5_ has increased by 27 °C and 11 °C, respectively, compared to neat PE. The position of PE and WS_2__Fe_2_O_3_ has not satisfactory influenced thermal stability of the nanocomposites. The lowest decrease of *T*_0.1_ and *T*_0.5_ was obtained for the composites with 2 wt% (11 °C lower than in pristine PE). *T*_0.1_ and *T*_0.5_ has increase by 8 °C and 16 °C, respectively, in the PE_WS_2_ with 2 wt% of WS_2_. Additionally, char yield increased of ∼7% in composite PE_WS_2__0.5% composite with PE_WS_2__Ni_2_O_3_ consisting 2 wt% exhibit the best value and it is increased ∼51% compared with PE. For materials of PE_WS_2__Fe_2_O_3_ the best value was obtained for 1 wt% and the value of char yield has not changed. Char yield and carbon monoxide generation of nanocomposites with metal oxides functionalized few layered WS_2_ are lower than the neat PE. We suppose that inorganic oxides assist in conversion of CO to CO_2_ and they limit generation of soot.^25^

**Table 1:** TGA data of nanocomposites

Sample	*T*_0.1_ [°C]	*T*_0.5_ [°C]	Char yield [%]
PE	292	424	1.89
PE_WS_2__0.5%	296	422	2.68
PE_WS_2__1%	307	417	0.76
PE_WS_2__2%	308	432	1.64
PE_WS_2__Fe_0.5%	292	394	0.74
PE_WS_2__Fe_1%	285	397	1.89
PE_WS_2__Fe_2%	281	413	1.48
PE_WS_2__Ni_0.5%	287	410	0.55
PE_WS_2__Ni_1%	281	389	0.57
PE_WS_2__Ni_2%	319	435	2.02

**Fig. 5:**
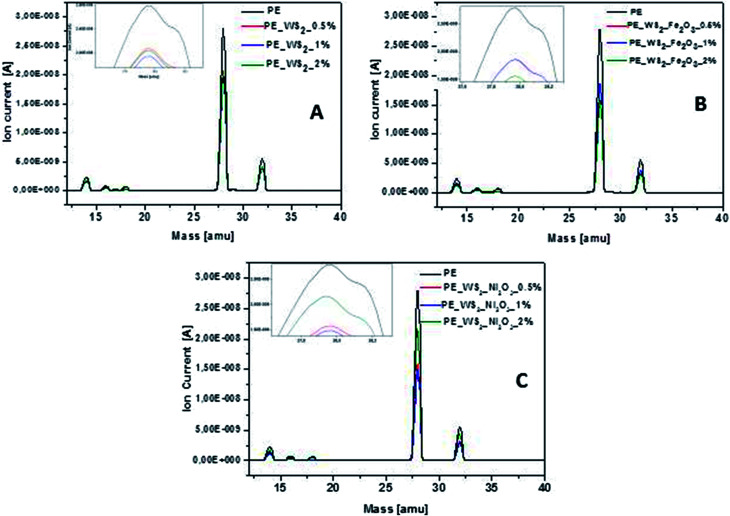
Mass spectrum of nanocomposites with: (A) WS_2_, (B) WS_2__Fe_2_O_3_ and (C) WS_2__Ni_2_O_3_.

During the thermogravimetric analysis, mass spectrometer was coupled and *in situ* gas analysis was carried out. The emission of toxic gases is considered as important parameter for flame-retardant materials. Mass spectra of all samples (Fig. 5) exhibit very significant peak at position of 28 amu, which corresponds to CO emission. The lowest value of carbon oxide emission was obtained for composite consisting PE_WS_2__Ni_2_O_3_ with 2 wt% compared to the polyethylene the value of emission decreases by 47%. Good efficiency of reduced CO emission was also obtained for PE_WS_2__Fe_2_O_3__2% (∼44% compared to pristine polyethylene). For composites with exfoliated WS_2_ the amount of filler has not significantly influenced the reduction CO emission. This value has decreased from 30% (for 2 wt%) to 35% (for 1 wt%) compared to polyethylene.

**Fig. 6:**
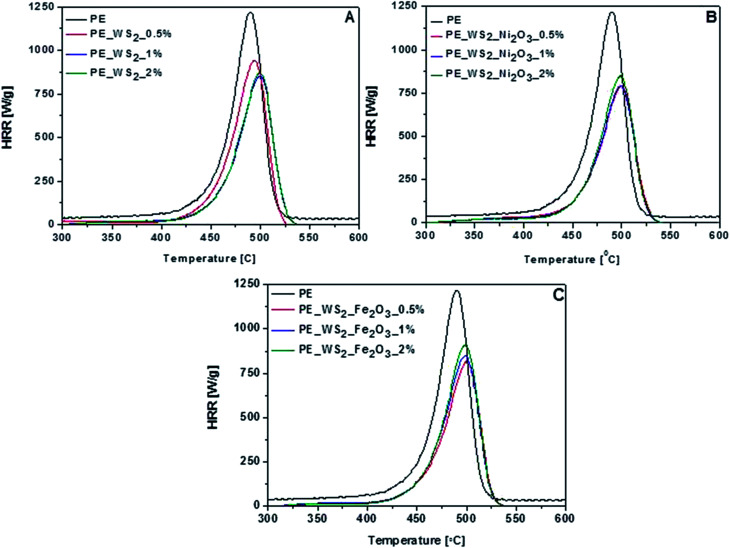
HRR curves of polyethylene with: (A) WS_2_, (B) WS_2__Fe_2_O_3_ and (C) WS_2__Ni_2_O_3_.

For assessment flammability properties of polyethylene-based nanocomposites microcalorimeter was used. The microscale combustion calorimeter (MCC) is a small-scale instrument that measures the heat release of a material by oxygen consumption calorimetry. Using this technique, the samples are exposed to a fast heating rate to mimic fire-type conditions. During MCC measurement several parameters are obtained, such as total heat release (THR), heat release rate (HRR) and heat release capacity (HRC). These parameters are crucial for assessing the fire risk of materials. The results of the MCC testing are summarized in Fig. 6 and Table 2.

**Table 2:** MCC data of nanocomposites

Sample	pHRR [W g^−1^]	THR [kJ g^−1^]	HRC [J g^−1^ K^−1^]
PE	1216	47	1179
PE_WS_2__0.5%	939	43.4	950
PE_WS_2__1%	852	39.9	858
PE_WS_2__2%	868	40.1	871
PE_WS_2__Fe_2_O_3__0.5%	819	38.8	824
PE_WS_2__Fe_2_O_3__1%	849	40.3	851
PE_WS_2__Fe_2_O_3__2%	910	41.1	907
PE_WS_2__Ni_2_O_3__0.5%	851	39.7	856
PE_WS_2__Ni_2_O_3__1%	799	38.3	796
PE_WS_2__Ni_2_O_3__2%	792	39	797

The HRR curves of nanocomposites derived from MCC are plotted in Fig. 6. The pHRR value has been often regarded as the most accurate indicator of flame. As it is shown, the addition of small amount of fillers leads to significant decrease of pHRR value in all composites. Compared to the PE, values of pHRR of composites with PE_WS_2_ and PE_WS_2__Fe_2_O_3_ have decreased with decreasing loading of fillers. The best improvement was obtained for composites consisting of 0.5 wt% of WS_2__Fe_2_O_3_ and it was about 33%. For composites consists PE_WS_2_ pHRR has decreased significantly compared with pristine PE. The largest decrease was observed for the sample containing 2 wt% of the filler. The value pHRR has decreased by 28%. Diez-Pascual *et al.* explains these improvements that WS_2_ could behave like a mass transport barrier which prevent the escape of volatile products generated during the burning and also hinders access to the matrix.^26^ The addition of fillers increase the temperature of maximum value peak HRR, which indicates the improvement in flame retardancy of nanocomposites. The most interesting value was obtained for composite the PE_WS_2__Ni_2_O_3_ containing 2 wt% of metal oxide. The peak heat release rate of PE_WS_2__Ni_2_O_3_ was reduced to 792 W g^−1^, and the total heat release was decreased to 39 kJ g^−1^. For almost each composite the temperature of pHRR increased by at least 10 °C. Interesting results was obtained also for PE_WS_2__Ni_2_O_3_ containing 1 wt% and 2 wt% the temperature has increased by 12 °C.

As is known to all, incorporation of 2D layered nanofillers usually increase the thermal stability of a polymer matrix due to the physical barrier effect which retards the diffusion of degradation products, gases and heat. MoS_2_ nanosheets must present better physical barrier effects compared to pristine PE. A lot of attention is devoted to MoS_2_ and its composite from PE,^27^ PS,^28^ PVA.^29^ However, composites with WS_2_ have much better fire retardancy and thermal stability properties. Compared data with MoS_2_ dispersed in the PE matrix, WS_2_ shows a decrease of flame retardancy. For the composites contained 2% nanofillers Ni_2_O_3_. The pHRR for composites with WS_2_ and MoS_2_ decreased ∼35% and 30%, respectively. The flame retardance analysis, it is reasonably speculated that independent WS_2_ nanosheets in the PE matrix act as nano-barriers to restrain the permeation of heat and oxygen and inhibit the effusion of volatile toxic materials. In comparison to the composites containing carbon nanotubes as fillers they did not exhibit flame retardant properties as good as WS_2_. Polyethylene with 2 wt% of CNT's decrease pHRR value about 24.5% compared to neat PE, but that nanocomposite reaches mu higher char yield.^30,31^

To measure the thermal conductivity of composites xenon flash method was used. The measurement was carried out along the thickness direction of each sample. The samples were coated with a thin layer of graphite to facilitate the absorption of the laser light at the surface of the sample. Measurements were conducted in the vacuum (1.0 × 10^−2^ bar). Three laser shots were applied to each sample at a room temperature. The final result for each parameter was presented as the average value of three partial measurements. The results are shown in the Table 3. Compared to the value of pristine polyethylene, thermal conductivity increased for all composites. The addition of small amount of filler brings the increase value of thermal conductivity about 100% for all the composites. Furthermore, the addition of 0.5 wt% of WS_2__Fe_2_O_3_ into PE exhibits the most significant increase in thermal conductivity. Compared to neat PE the value has increased about ∼240%. The most significant difference of value for composites containing WS_2__Ni_2_O_3_ was obtained for 2 wt% and it was 290% higher than for pure PE. The interesting value was obtained for composite PE_WS_2__1%. In this case, the improvement of thermal conductivity was 230% compared to pristine PE.

**Table 3:** Thermal conductivity of composites

Sample	Thermal conductivity [W m^−1^ K^−1^]
PE	186
PE_WS_2__0.5%	370
PE_WS_2__1%	524
PE_WS_2__2%	507
PE_WS_2__Ni_2_O_3__0.5%	427
PE_WS_2__Ni_2_O_3__1%	510
PE_WS_2__Ni_2_O_3__2%	550
PE_WS_2__Fe_2_O_3__0.5%	454
PE_WS_2__Fe_2_O_3__1%	389
PE_WS_2__Fe_2_O_3__2%	433

## 4. Conclusion

In this study, the aqueous phase exfoliated WS_2_ nanosheets were successfully functionalized with metal oxides (nickel and iron) and incorporated into polyethylene matrix by extruder melting. Adding small amount of layered nanofillers improved the thermal stability and fire resistance of composites significantly. MCC, TGA and thermal conductivity measurements indicate that the reduction of flammability is dependent on the content of WS_2_ fillers. The best improvement in thermal degradation was obtained for PE_WS_2__Ni_2_O_3__2% for which the temperature *T*_0.1_ and *T*_0.5_ increased by 27 °C and 11 °C, respectively, compared to neat PE. Significant decrease in value of peak heat release rate and CO emission was obtained for PE_WS_2__Ni_2_O_3__0.5% which was 33% and 44%, respectively. Thermal conductivity of this composite has increased by 144% compared to pristine polyethylene. The reduction of fire hazard was attributed to the physical barrier effect of WS_2_. Therefore, it is believed that new flame-retardant nanocomposites can find important and practical applications, such as wall insulation, cable ropes, pipes or casings.

## Conflicts of interest

There are no conflicts to declare.

## Supplementary Material
